# Evaluation of the Effectiveness of Acupuncture in the Treatment of Knee Osteoarthritis: A Case Study

**DOI:** 10.3390/medicines5010018

**Published:** 2018-02-05

**Authors:** Joana Teixeira, Maria João Santos, Luís Carlos Matos, Jorge Pereira Machado

**Affiliations:** 1 ICBAS- Institute of Biomedical Sciences Abel Salazar, University of Porto, 4050-313 Porto, Portugal; joanabcteixeira@gmail.com (J.T.); mjrs.mtc@gmail.com (M.J.S.); 2 Faculdade de Engenharia da Universidade do Porto, Rua Dr. Roberto Frias, s/n 4200-465 Porto, Portugal; lcmatos@fe.up.pt; 3 LABIOMEP—Porto Biomechanics Laboratory, University of Porto, 4200-450 Porto, Portugal

**Keywords:** osteoarthritis, acupuncture, Heidelberg Model of Traditional Chinese Medicine

## Abstract

**Background:** Osteoarthritis is a widespread chronic disease seen as a continuum of clinical occurrences within several phases, which go from synovial inflammation and microscopic changes of bone and cartilage to painful destructive changes of all the joint structures. Being the most common joint disease, it is the leading cause of disability in working individuals above 50 years of age. In some cases, conventional treatments produce just a mild and brief pain reduction and have considerable side-effects. Contemporary Traditional Chinese Medicine (TCM) is a model of systems biology based on a logically accessible theoretical background. It integrates several therapeutic approaches, among them acupuncture, which has shown effective results in the treatment of knee and hip osteoarthritis, minimizing pain, improving functionality and consequently leading to a better quality of life. **Methods:** The present case study included two patients with clinical signs of osteoarthritis and diagnosis of medial pain, as defined by the Heidelberg Model of TCM. Over 6 weeks, those patients were treated with acupuncture, with a frequency of one session a week. The sessions lasted for thirty minutes and were based on the needling of 4 local acupoints. Before and after each session, pain and mobility assessments were performed. **Results:** The results were positive, with significant reduction of pain and increased knee joint flexion amplitude and mobility. **Conclusion:** Acupuncture was effective as an alternative or complementary treatment of knee osteoarthritis, with high levels of improvement within a modest intervention period.

## 1. Introduction

Osteoarthritis is a chronic degenerative joint disease demonstrating articular cartilage damage and leading to disabling pain and joint dysfunction [1]. Clinically, osteoarthritis is characterized by pain, typically with gradual onset that worsens over time, swollen joint caused by synovitis, morning stiffness, crackling bone, muscle atrophy, narrowing of the intra-articular space, osteophyte formation, subchondral bone sclerosis and cyst formation [2].

Osteoarthritis of the knee is a multifactorial disease, whose etiology includes generalized systemic disease (e.g., gout, rheumatoid arthritis), constitutional factors (e.g., age, gender and genetics) and also biomechanical factors (e.g., joint damage, muscle weakness, overweight and obesity) [3]. The higher incidence and severity in females is related to hormonal status. Fluctuations of sex hormone levels in young females and loss of ovarian sex hormone production due to menopause in older ones contribute to the observed differences in gender prevalence [4,5].

The treatment of knee osteoarthritis may be categorized as either conservative or surgical. Conservative treatment may include medication, manual and physical therapies, medially directed patellar taping, walking aids, thermal agents, weight loss, Tai Chi practice, swimming, water aerobics, and resistance exercises. The American College of Rheumatology recommends most of the previous non-pharmacological approaches in the initial treatment of this disease [6]. Nevertheless, common interventions involve the use of analgesics, anti-inflammatories and infiltrations with corticosteroids. Pharmacological intervention may include the use of glucosamine, glucosamine with chondroitin, chondroitin, acetaminophen, oral and topical NSAIDs, tramadol, and intraarticular corticosteroid injections, intraarticular hyaluronate injections, duloxetine, and opioids [7]. Physiotherapy, occupational therapy and psychomotor rehabilitation may help controlling pain and improving joint function, restoring quality of life [6,8]. Surgical treatment, which can be divided into joint-preserving, such as arthroscopy and osteotomy (femoral or tibial), and joint-replacing procedures, such as partial and total arthroplasties, should be indicated in cases without symptom relief after conservative approaches [9].

Although conventional approaches, either pharmacological and surgical have been reported as successful, side effects such as toxicity manifestations, complications involving anesthesia, infection, osteoligamentous surgery-induced injuries are issues that cannot be ignored [1]. In this scenario, acupuncture comes as an alternative therapeutic approach in knee osteoarthritis conditions, showing good results in pain reduction and rehabilitation of motor abilities, improving functionality and quality of life, either when used as a stand-alone therapy or together with other procedures [7,10,11,12,13,14,15,16,17,18,19].

Nowadays, acupuncture is accepted among the Portuguese medical community with regard to its mechanisms of action as a medical procedure, with its analgesic and anti-inflammatory effects recognized a long time ago by the World Health Organization, and is considered superior to placebo in published randomized controlled trials [20]. Indeed, this integration requires a science-based approach supported by controlled research. Nevertheless, even with studies corroborating its benefits, there is still a great debate within the scientific community with regard to its mechanisms of action and therapeutic methodology [21,22,23].

Eastern and Western therapeutic approaches and anatomical and physiological views of the knee region are considerably different. In TCM, the knee is sustained by the liver (hepatic) and by the conduits that pass through this region; namely, the stomach, gallbladder and bladder, known as the *yang* conduits, and the liver, kidney and Spleen, known as the *yin* conduits [24,25,26]. These conduits are involved in the neurovegetative patterns of that anatomical region acting on the knee joint, nerves, and being responsible for the normal functioning of the knee processes [25]. The most common form of knee pain, known in TCM as center or medial pain, is characterized by a localized pain in the medial part of the knee that the Spleen conduit passes through [27]. Although many other acupoints could be used in the treatment of this condition [17], the approach based on the Heidelberg Model of TCM involves supporting the Centre by the simultaneous stimulation of specific acupoints from the Spleen and Stomach conduits, which belong to the Phase Earth, in order to promote orthopathy [26].

In this case study, conducted with two patients suffering from knee osteoarthritis with constant pain, the research team aimed to evaluate the effectiveness of acupuncture as a treatment procedure, using a protocol based on the needling of just 4 local acupoints to treat pain and increase joint mobility.

## 2. Materials and Methods

The patients considered in this study were selected according to the following inclusion and exclusion criteria.

**Inclusion criteria:** clinical signs of osteoarthritis and diagnosis of medial pain, as defined by Heidelberg Model of TCM, knee pain, mobility commitment and impairment in daily living activities, such as going up and down stairs.

**Exclusion criteria:** pregnancy and lactation, psychiatric or neurological disorders, presence of inflammatory autoimmune disease, history of substance abuse.

### 2.1. Sample Characterization

Subject A—A female of 46 years old, formerly a cook in a private hospital, currently unemployed, with acute pain in the knee and signs of osteoarthritis detected by imaging tests. Acupuncture was performed after a month of intense pain and loss of mobility. The patient used crutches during the rehabilitation period.

Subject B—A female of 56 years old, formerly a secretary, currently unemployed, suffering from internal and femoro-patellar gonarthrosis, was victim of a stairs accident in December 2010 and submitted to two surgical interventions in 2010 and 2012. The patient took medication and decided to start acupuncture after two years of constant pain and mobility problems.

A written informed consent from patients was required to proceed with the intervention.

### 2.2. Materials

Single-use sterilized acupuncture needles (25 mm length and 0.25 mm diameter) brand Tewa, Goniometer, and Portuguese pain assessment scale.

### 2.3. Methodology

The intervention consisted of 6 thirty-minute acupuncture sessions with a frequency of one session a week. The degree of pain was evaluated before and after the treatment by using the numeric pain rating scale, which is a single-dimensional instrument to assess pain intensity at rest or with activity. This scale is frequently used in adults with chronic pain due to rheumatic diseases, and is scored by choosing a number between 0 (no pain) and 10 (extreme pain or other label) to rate current pain intensity [28]. The mobility range tests were performed with a Goniometer at the beginning and 10 min after each session.

The acupuncture treatments were performed by a qualified TCM practitioner and the points used in this study were: St34 *Liang Qiu* (S34 *Monticulus Septi*)*,* St36 *Zu San Li* (S36 *Vicus Tertius Pedis*), Sp9 *Yin Ling Quan* (L9 *Fons Tumuli Yin*) and Sp10 *Xue Hai* (L10 *Mare xue*) (acupoints denomination according to the Heidelberg Model of TCM is quoted between bracelets).

## 3. Results

**Figure 1:**
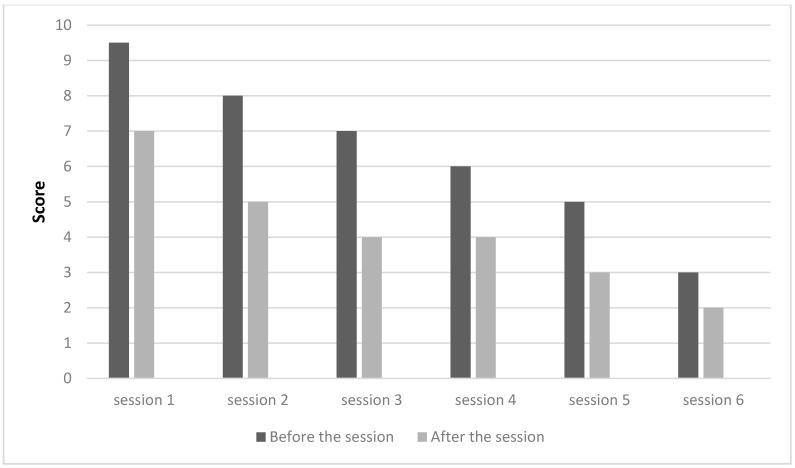
Assessment of pain per session, subject A.

**Figure 2:**
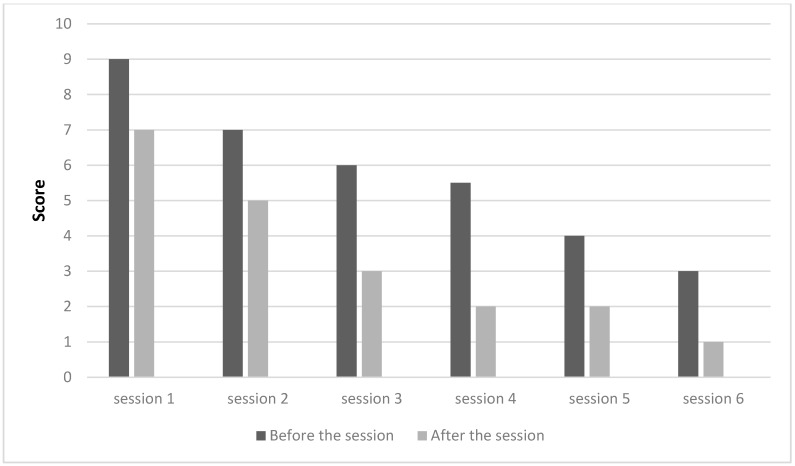
Assessment of pain per session, subject B.

As shown in Figure 1 and Figure 2, subjects A and B reported clear improvements in their condition, with pain reduction along the acupuncture treatment (pain scores dropped from 9.5 to 2 and from 9 to 1 in subjects A and B, respectively). These good results allowed them to stop taking conventional medication for pain. The analgesic effect of acupuncture has been often reported by other authors. This effect can be induced both by a local stimulation of the tissue, thus resulting in the release of inflammatory related substances, vasodilatation and the increase of serotonin and immune cells, as well as by a hypothalamus activation and related endorphins release [29].

Although some studies point to pain relief in patients suffering from osteoarthrosis, the effects on articular mobility are unclear, probably due to different types of therapeutic methodologies [16,30]. As standardization is a key issue in research, protocols must control a wide range of variables, such as the acupuncture technique, the intervention protocol, the duration of the treatment, the selected acupoints, just to mention a few. It is easy to find studies in which a vast selection of acupoints and different stimulations were used in the treatment of knee osteoarthritis. Some used a combination of 9 to 10 local and distal points from several conduits such as Stomach, Spleen, Gall Bladder, Kidney, Urinary Bladder, Liver, Large Intestine, and the *xiyan* extraordinary acupoint, as well as a combination of manual and electrical stimulation [19,31,32,33,34,35]. In these studies, the duration of the intervention and the frequency of the sessions also varied between 2 and 12 weeks with one or two sessions a week. In fact, methodological standardization is a hard task, not only because, in some cases, there is no consensus between practitioners and their diagnosis, but also because each patient has a specific homeostatic imbalance and physiological compensation when submitted to the same stimulus [36].

In our study, Subjects A and B presented an increase in mobility and range of motion following treatment. The degree of knee flexion raised up from 45° with pain to 160° without pain to subject A and from 90° to 160° to subject B (Table 1). In this process, subject A abandoned the use of crutches.

**Figure 3:**
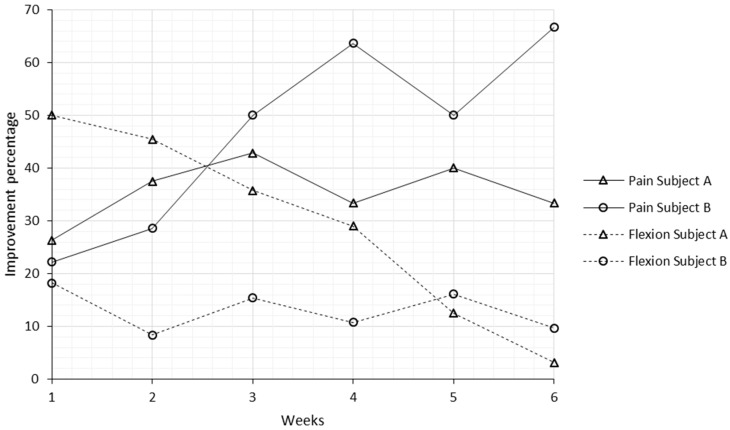
Improvement percentages for pain and knee joint flexion amplitude per session, considering each session initial and final scores, along the experimental period.

The improvement percentages for pain and knee joint flexion amplitude per session, considering each session initial and final scores, along the experimental period are shown in Figure 3.

Regarding pain, Subject A experienced an increasing tendency in the improvement percentage per session from week 1 to 3, and from this point on, this tendency stopped rising. The average improvement percentage per session for Subject A was 36%, and the overall improvement at the end of the experimental period reflecting the initial to final evaluation, was 79%. In the case of Subject B, with the exception of week 5, the improvement per session was always higher than the week before, with an average of 47% and overall improvement of 89%. One week after each treatment, Subjects A and B had average regressions on pain of 19% and 29%, respectively, taking into account the previous evaluation and the evaluation before the acupuncture session.

With regard to knee joint flexion, the condition of Subject A in the beginning of the experimental period was worse than Subject B. Subject A experienced an improvement of 50% immediately after the first session. The following weeks, a decreasing tendency was noticed as knee flexion, before each session, tended to be higher as a result of the patient’s response to the treatment. The average per-session and overall improvement percentages were 29% and 72% for Subject A and 13% and 42% for Subject B. Considering that Subject B’s condition was better, the improvements per session were not as high as Subject A, and remained almost stable along the experimental period. Regressions one week after each session were also noticed in knee flexion amplitude, with scores falling on average 17% and 6%, for Subjects A and B, respectively.

The overall analysis of pain and knee flexion improvements presented in this case study is highly satisfactory. High improvement percentages, an average of 84% for pain and 57% for join flexion mobility, were achieved within a modest intervention period, 6 weeks, with just one session a week, and with a reduced number of needled acupoints, compared to most of the treatment protocols used in other studies. Knee flexion mobility per session (an average of 21%) was higher than the outcomes reported in similar studies (9% [30] and 16% [37]). Although the beneficial effects of acupuncture are shown here in just two patients, and the small sample can skew results, our study supports the thesis that acupuncture can be used as an effective therapeutic tool in the treatment of knee osteoarthritis.

## 4. Conclusions

Contemporary understanding of TCM considers it as a model of systems biology with a holistic therapeutic approach, in which the diagnosis is key to achieving positive results. Thus, systematized science-based analysis and decisions, both of the pathological condition and therapeutic intervention, are required to validate the results obtained by the use of acupuncture as a therapeutic tool. In our study, within the framework of the Heidelberg Model of TCM, we found that acupuncture was effective in the treatment of knee osteoarthritis with significant pain relief and improvement in knee joint mobility and range in 2 subjects whose initial presentation was characterized by extreme pain, scored 9.5 and 9, as well as reduced joint flexion amplitude, 45° and 90°. The overall pain relief, considering the whole intervention period, was 79% and 89% for subject A and B, respectively. With regard to mobility and range of motion, positive results were also found with considerable improvements in the knee flexion angle from 45° to 160° in subject A (an improvement of 72%), and 90° to 155° in subject B (an improvement of 42%). Although regressions, both in pain and joint flexion mobility, were noticed one week after each treatment, the improvements in each session were high enough to overcome this issue, allowing for maintenance of the recovery with increasing tendency along the experimental period.

In addition to concluding that acupuncture was effective as a therapeutic tool with high levels of recovery when compared with similar published studies, it is important to mention that these results were achieved within a modest intervention period, 6 weeks, with just one session a week, and with a reduced number of needled acupoints, compared to most of the treatment protocols used in other studies, and doesn’t require electrical stimulation. Our outcomes are a good example of a successful intervention that can contribute to the debate about methodological standardization of acupuncture in the treatment of knee osteoarthrosis.

We are aware of the limitations of this study and suggest a follow up study with a statistically representative sample.

## Figures and Tables

**Table 1:** Knee joint flexion amplitude before and after acupuncture sessions.

Session	Subject A		Subject B	
	Before	After	Before	After
1st	45°	90°	90°	110°
2nd	60°	110°	110°	120°
3rd	90°	140°	110°	130°
4th	110°	155°	125°	140°
5th	140°	160°	130°	155°
6th	155°	160°	140°	155°
